# Fluid Overload and Kidney Injury Score as a Predictor for Ventilator-Associated Events

**DOI:** 10.3389/fped.2019.00204

**Published:** 2019-05-22

**Authors:** Jarin Vaewpanich, Ayse Akcan-Arikan, Jorge A. Coss-Bu, Curtis E. Kennedy, Jeffrey R. Starke, Satid Thammasitboon

**Affiliations:** ^1^Division of Pediatric Critical Care Medicine, Department of Pediatrics, Ramathibodi Hospital, Mahidol University, Bangkok, Thailand; ^2^Section of Critical Care Medicine, Baylor College of Medicine and Texas Children's Hospital, Houston, TX, United States; ^3^Department of Pediatrics, Baylor College of Medicine and Texas Children's Hospital, Houston, TX, United States; ^4^Section of Nephrology, Baylor College of Medicine and Texas Children's Hospital, Houston, TX, United States; ^5^Section of Infectious Disease Medicine, Baylor College of Medicine and Texas Children's Hospital, Houston, TX, United States

**Keywords:** ventilator associated pneumonia, ventilator-associated condition, infection-related ventilator-associated complications, FOKIS, organ cross-talk

## Abstract

**Objective:** The Pediatric and Neonatal Working group developed new ventilator associated events (VAE) definitions for children and neonates. VAE includes ventilator-associated condition (VAC), infection-related ventilator-associated complication (IVAC), and ventilator-associated pneumonia (VAP). Acute kidney injury (AKI) and fluid overload (FO) have been associated with worse clinical outcomes of ventilated children. Fluid Overload and Kidney Injury Score (FOKIS) is an automatically calculated score that combines AKI and FO in one numeric quantifiable metric. This study analyzed the association between FOKIS and VAE.

**Design:** Retrospective matched case control study.

**Setting:** A freestanding children's hospital.

**Patients:** A total of 168 who were ventilated > 2 days.

**Interventions:** None.

**Measurements and Main Results:** We identified 42 VAC cases (18 IVAC and 24 non-infection-related VAC cases). Controls were matched to cases for age, immunocompromised status and ventilator days prior to VAC. VAC cases had longer ICU days, median (IQR), 28.5 (15, 47) vs. controls 11 (6, 16), *p* < 0.001; longer ventilation days, 19.5 (13, 32) vs. 9 (4,13), *p* < 0.001; and higher hospital mortality, 45.2 vs. 18%, *p* < 0.001. VACs had a higher incidence of AKI, 85.7 vs. 47.3%, *p* < 0.001; higher peak daily FO% within 3 days preceding VAC, mean (SD), 8.1(7.8) vs. 4.1 (3.4), *p* < 0.005; and higher peak FOKIS, 6.4(3.8) vs. 3.7(2.8), (*p* < 0.001). Multivariate regression model adjusted for severity of illness identified peak FOKIS (odds ratio [OR] 1.29, 95%CI: 1.14–1.48, *p* < 0.001) and peak inspiratory pressure (OR 1.08, 95%CI: 1.02–1.15, *p* = 0.007) as risk factors for VAC.

**Conclusions:** The FOKIS and its clinical variables were associated risk factors for ventilator-associated events. Further studies will determine the utility of FOKIS as a predictor for VAEs.

## Introduction

The Centers for Disease Control and Prevention (CDC) replaced its national surveillance definition of ventilator-associated pneumonia (VAP) in adults with definitions for ventilator-associated events (VAEs) which included ventilator-associated condition (VAC), infection-related ventilator-associated complication (IVAC) and ventilator-associated pneumonia (VAP) (1, 2). We applied the adult definition to children admitted to a pediatric intensive care unit (PICU) and determined VAC as one of the predictors of hospital mortality (3). In addition, we identified immunocompromized status, tracheostomy dependence, and chronic respiratory disease as risk factors for the VAC occurrence (3).

Fluid overload (FO) has been shown to be associated with respiratory morbidity in critically ill children. The peak cumulative FO is associated with impaired oxygenation in mechanically ventilated children (4) and the extent of FO at 48 h of PICU admission is associated with higher oxygenation index and increased number of ventilator days (5). Fluid overload also has been found to be a major risk factor for developing VAC in adults (6) and depletive fluid-management strategy is associated with a lower incidence of VAP (7). We recently identified in a matched case control study that severity of FO was associated with VAC occurrence (8).

Acute kidney injury (AKI) has been associated with acute lung injury (ALI) (9–13). Our previous study revealed AKI defined by Pediatric Risk, Injury, Failure, Loss, End Stage Renal Disease (pRIFLE) score as a risk factor for the occurrence of VAC and IVAC (8). Fluid Overload Kidney Injury Score (FOKIS) is a scoring system that incorporates FO severity and nephrotoxic medication exposure to AKI assessment by pRIFLE. FOKIS has been shown to be independently associated with ICU mortality and length of stay, and had better correlation with mortality than FO or AKI definition alone (14).

The Pediatric and Neonatal VAE Working Group recently released the new VAC definition for children based on an increase in minimum daily FiO_2_ by at least 0.25 or mean airway pressure (Paw) by at least 4 cm H_2_O for 2 or more days after a period of stability (15). We hypothesized that FOKIS and/or its clinical variables are associated with the occurrence of VAE.

## Materials and Methods

We conducted a retrospective matched case-control study among consecutive patients admitted to the Pediatric Intensive Care Unit (PICU) during October 2012–March 2014 at Texas Children's Hospital, Houston, TX. Patients placed on conventional mechanical ventilation or high frequency oscillatory ventilation (HFOV) for >2 days were considered for inclusion. Patients excluded if: admitted to cardiac or neonatal ICU, received extracorporeal membrane oxygenation within the first 3 days of PICU admission; had a tracheostomy and were ventilator dependent; were receiving chronic renal replacement therapy (RRT); or died within 3 days of intubation. We applied the pediatric VAC definition to identify patients with VAC (case) and matched these patients 1:3 to patients without VAC (control) on the basis of age, immunocompromized status and duration of mechanical ventilation prior to the VAC occurrence. For duration of mechanical ventilation, total ventilator days in the control group was matched to the case patient's time from intubation to VAC occurrence.

## Definitions

### Ventilation-Associated Events

We developed a VAC module according to pediatric VAC criteria (i.e., sustained (> 2 days) increases in ventilator FiO_2_ > 0.25 or mean airway pressure (Paw) >4 cm H_2_O after at least 2 days of stability), and applied the VAC module to the local PICU surveillance using FOKIS to identify patients who have risk of renal injury. Once a VAC case was identified, we evaluated the probability of IVAC and Possible Ventilator-Associated Pneumonia (PVAP) based on the CDC definitions for adults (2).

### Fluid Overload Kidney Injury Score (FOKIS)

The FOKIS comprises three variables; (1) AKI severity according to pRIFLE, (2) FO severity, and (3) nephrotoxic medication exposure (Supplementary Material Table 1). This scoring system serves as a decision support tool for identification of patients with renal disease burde (14) at our institution.

The AKI severity was calculated by assigning 1, 2, or 3 points for pRIFLE according to creatinine and urine output criteria (16). We calculated estimated creatinine clearance (eCCl) by using the Schwartz formula (17) and baseline eCCl was calculated using the lowest serum creatinine in the 3 months preceding the admission. Patients without a known baseline creatinine were assumed to have normal renal function and were automatically assigned a baseline eCCl of 120 ml/min/1.73 m^2^ (18). For FO severity, we calculated cumulative %FO based on the formula: [(fluid in (ml) – fluid out (ml) from PICU admission to date of VAC event) / PICU admission weight (kg), and x 100%]. FO severity contributed to 1 to 5 points for the extent of FO <15% in 5% increments. The third variable of the score is exposure to nephrotoxic medications. The minimum of three different nephrotoxic medications was considered significant exposure contributing 1 point, and each additional nephrotoxic medication exposure added one point toward the total score. The list of potentially nephrotoxic medications is shown in Supplementary Material Table 2. The FOKIS was automatically calculated by our clinical decision support system each time a new dependent data was added to the electronic medical record (EMR), or when there was a change in any of the parameters (i.e., when a new medication was added or when AKI stratum changed).

## Data collection

We reviewed each patient's EMR to identify age, sex, body weight, Pediatric Risk of Mortality (PRISM) 3 score and Pediatric Index of Mortality (PIM) 2 score on PICU admission, ventilator settings: peak inspiratory pressure (PIP) and mean airway pressure (Paw), microbiology results, and radiographic findings. Cumulative %FO was calculated and the peak value was used in FOKIS for grading. We also calculated daily %FO for 3 days preceding the VAC event as the difference between daily total input and output / PICU admission weight in Kg times 100%. The highest value of daily %FO within 3 days preceding the VAC was chosen as an independent variable for analysis. Clinical outcomes collected included duration of mechanical ventilation, length of stay (LOS) in PICU, hospital LOS, and PICU mortality. We retrieved the peak FOKIS from the PICU admission to the time of VAC and peak FOKIS within 3 days preceding the event for each case, and retrieved the same variables from the matched time period in each matched control. This study was approved with a waiver of informed consent by the institution review board at the Baylor College of Medicine.

## Data Analysis

We compared cases with controls for demographics and clinical variables. We first analyzed the VAC cases and their matches, and then performed repeat analysis for IVAC cases and their matches. Descriptive data are presented as means with standard deviation (SD) or medians with interquartile range (IQR) for continuous variables and frequencies with percentages for categorical variables. When appropriate, we used non-parametric tests, the Mann-Whitney and Fisher exact tests, to compare continuous and categorical variables, respectively. We performed univariate conditional logistic regression analyses examining the associations between each covariate with VAC and IVAC, respectively. We ranked covariates based on their *p*-values from the likelihood ratio test and then performed separate multivariable conditional logistic regression analyses for VAC and IVAC. We included all variables with *p*-values less than or equal to 0.10 in the multivariate analyses. We set to include PIM 2 score in the models *a priori* in order to adjust for severity of illness. All analyses were performed using Stat View (SAS Institute, Inc., Cary, NC, USA).

## Results

Of a total of 2,830 patients admitted to the PICU during the study, 1,358 patients received mechanical ventilation (7,863 ventilator days); 730 patients were ventilated for more than 2 days. Of these, 66 patients met the VAC definition. The VAC incidence was 8.4 per 1,000 ventilator days. Twenty-four patients were excluded due to: a tracheostomy with chronic ventilator dependency (23 patients), and chronic kidney disease requiring chronic peritoneal dialysis (1 patient). Of the 42 patients included, 18 patients met the IVAC criteria definition for an incidence of 2.2 per 1,000 ventilator days. Nine patients were classified as PVAP and 9 had unidentified infection. There were 24 patients classified as VAC from non-infectious causes: atelectasis (33%), pulmonary edema (25%), pulmonary hemorrhage (13%), pneumothorax (4%), acute respiratory distress syndrome (4%), worsening pulmonary hypertension (8%) and unknown cause of VAC (13%).

The characteristics of VAC and IVAC cases compared with controls are shown in Table 1. There were no differences in disease severity score between cases and controls. Cases had more severe AKI prior to event day. Considering the 3 days preceding the VAC, the peak daily %FO was significantly higher in the case vs. control group; there was no difference in the peak cumulative %FO from PICU admission. Comparing clinical outcomes, VAC and IVAC cases had significantly more total ventilator days, longer PICU and hospital LOS, and higher PICU mortality than controls.

**Table 1:** Characteristics of patients with ventilator-associated conditions, infection-related ventilator-associated complications, and their matched controls.

**Variable**	**Ventilator-associated condition (VAC) cases (*n* = 42)**	**Controls (*n* = 126)**	***P*-value**	**Infection-related ventilator-associated complication (IVAC) cases (*n* = 18)**	**Controls (*n* = 54)**	***P*-value**
**PATIENT CHARACTERISTIC**						
Age, yr, median (IQR)	1.22 (0.48, 10.4)	1.36 (0.41, 9.9)	0.896	2.02 (0.69, 8.6)	1.92 (0.69, 10.9)	0.797
Sex, male, n (%)	21 (50%)	69 (54.8%)	0.592	12 (66.7%)	56 (51.9%)	0.310
Body weight, kg, median (IQR)	9.8 (6.5, 28.4)	10.1 (6.9, 27.6)	0.490	10.3 (6.8, 28.3)	12.3 (8.2, 34.1)	0.725
Immunocompromised status	; (38.1%)	42 (33.3%)	0.574	9 (50%)	42 (38.9%)	0.440
PRISM3 score, mean(SD)	11.41 (7.99)	9.32 (8.39)	0.161	8.78 (6.37)	9.62 (8.66)	0.694
PIM2 score, mean (SD)	−2.79 (1.82)	−3.27 (1.58)	0.109	−2.78 (1.89)	−3.19 (1.64)	0.334
**KIDNEY AND FLUID OVERLOAD VARIABLES**						
AKI severity, pRIFLE, *n* (%)						
Risk = 1	6 (14.3%)	30 (23.3%)	<0.001	3 (16.7%)	27 (25%)	<0.001
Injury = 2	17 (40.5%)	19 (15.1%)		8 (44.4%)	16 (14.8%)	
Failure = 3	13 (31%)	12 (9.5%)		6 (33.3%)	12 (11.1%)	
RRT, n (%)	8 (19%)	11 (8.7%)	0.067	2 (11.1%)	10 (9.3%)	0.681
Peak daily %FO within 3 days preceding event, mean (SD)	8.12 (7.78)	4.06 (3.38)	0.002	8.52 (8.85)	3.88 (3.40)	0.042
Peak cumulative %FO, mean (SD)	29.0 (25.54)	22.6 (14.58)	0.130	35.1 (29.22)	22.2 (15.17)	0.085
**FOKIS VARIABLES**						
Peak FOKIS since PICU admission mean (SD)	6.36 (3.75)	3.72 (2.84)	<0.001	6.72 (3.88)	3.83 (2.93)	0.007
Peak FOKIS within 3 days preceding event mean (SD)	4.98 (3.68)	2.15 (2.57)	<0.001	5.17 (4.18)	2.19 (2.70)	0.009
**VENTILATOR PARAMETERS VARIABLES**						
Mean PIP, mean (SD)	28.42 (7.75)	24.41 (6.33)	0.002	31.46 (7.43)	24.63 (6.20)	<0.001
Mean Paw, mean (SD)	15.64 (4.89)	11.73 (3.75)	<0.001	15.76 (4.35)	11.91 (3.94)	<0.001
AUC Paw	93.5 (58-210)	92.2 (48-154)	0.133	106.9 (72-190)	100.4 (62-157)	0.397
**OUTCOME VARIABLES**						
Ventilator days, days, median (IQR)	19.5 (13, 32)	9 (4, 13)	<0.001	19.5 (14, 32)	9.5 (4, 14)	0.021
PICU LOS, days, median (IQR)	28.5 (15, 47)	11 (6, 16)	<0.001	28 (19, 43)	11 (6, 16)	0.007
Hospital LOS, days, median (IQR)	42.5 (21, 92)	24.5 (13, 45)	0.006	43.5 (32, 97)	27 (14, 47)	0.016
Mortality, *n* (%)	19 (45.2%)	18 (14.3)	<0.0001	7 (38.9%)	7 (12.9%)	0.034

*AKI, Acute Kidney Injury; PRISM 3, Pediatric risk of mortality; PIM 2, Pediatric index of mortality; pRIFLE, Pediatric Risk, Injury, Failure, Loss, End-Stage Renal Disease; RRT, Renal replacement therapy; FO, fluid overload; FOKIS, Fluid Overload and Kidney Injury Score; PIP, Peak inspiratory pressure; Paw, Mean airway pressure; AUC, Area under the curve; PICU, Pediatric Intensive Care Unit; LOS, Length of stay; IQR, Interquartile Range. Statistical analysis by Mann-Whitney for continuous variables and Fisher's exact test for categorical variables*.

On univariate regression analyses (Table 2), patients who had more severe AKI, higher %FO, higher FOKIS and higher ventilator support requirement were more likely to develop VAC. The univariate regression analysis for IVAC showed similar results.

**Table 2:** Univariate risk factor analysis for ventilator-associated conditions (VAC) and infection-related complications (IVAC).

**Variables**	**VAC**		***P*-value**	**IVAC**		***P*-value**
	**OR**	**95%CI**		**OR**	**95%CI**	
AKI Severity (pRIFLE)	2.46	1.72–3.52	<0.001	2.72	1.62–4.56	<0.001
Peak daily %FO within 3 days preceding event	1.18	1.08–1.29	<0.0005	1.19	1.05–1.34	0.005
Peak accumulative %FO	1.02	1.00–1.04	0.052	1.03	1.01–1.06	0.010
Peak FOKIS since PICU admission	1.26	1.13–1.40	<0.001	1.26	1.09–1.45	0.001
Peak FOKIS within 3 days preceding event	1.32	1.17–1.49	<0.001	1.29	1.11–1.49	0.001
Mean PIP	1.09	1.03–1.15	0.003	1.16	1.07–1.27	0.001
Mean Paw	1.21	1.11–1.32	<0.001	1.19	1.07–1.32	0.001
AUC Paw	1.00	1.00–1.01	0.070	1.00	0.99–1.01	0.181
PIM2 score	1.18	0.96–1.45	0.116	1.14	0.87–1.50	0.335

*VAC, Ventilator associated conditions; IVAC, Infection-related Ventilator-Associated Complications; AKI, Acute Kidney Injury; pRIFLE, Pediatric Risk, Injury, Failure, Loss, End-Stage Renal Disease; FO, Fluid overload; FOKIS, Fluid Overload and Kidney Injury Score; PIP, Peak inspiratory pressure; Paw, Mean airway pressure; AUC, Area under the curve; PIM2, Pediatric Index of Mortality*.

**Figure 1:**
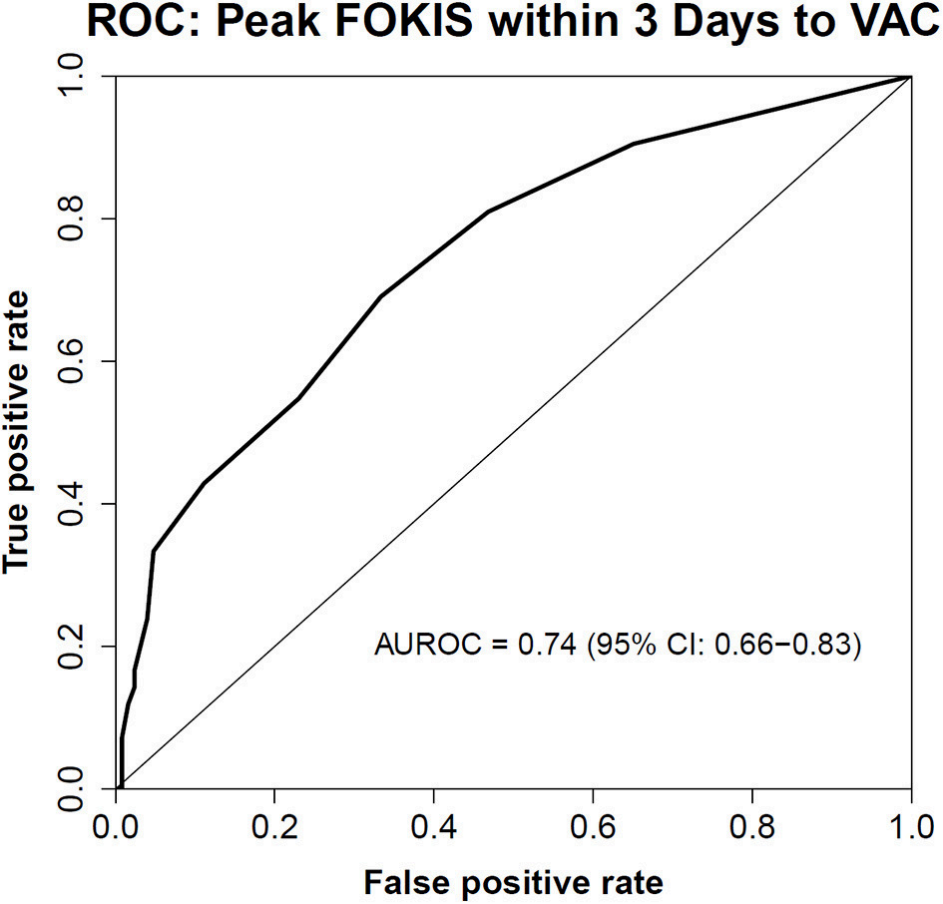
The Receiver Operating Characteristic (ROC) Curve of the peak FOKIS within 3 days and VAC occurrence. An area under the ROC curve is 0.74 (95% CI, 0.65–0.83; *p* < 0.001).

**Figure 2:**
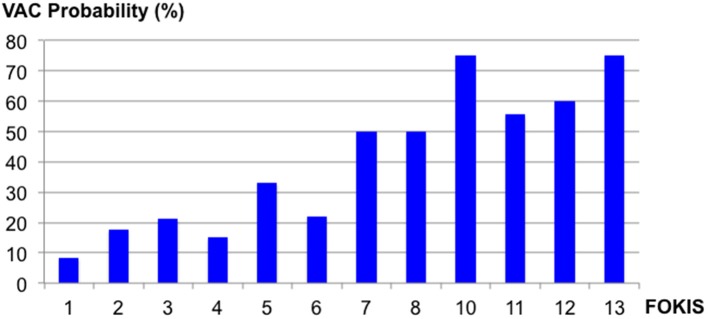
The probability of the Ventilator-Associated Condition (VAC) occurrence according to different Fluid Overload and Kidney Injury Scores (FOKIS).

In a multivariable logistic regression model (Table 3), the independent risk factors for VAC included, AKI severity (OR, 2.15; 95%CI, 1.39–3.31), peak %FO within 3 days preceding the VAC (OR, 1.22; 95% CI, 1.08–1.37) and mean PIP (OR, 1.09; CI, 1.02–1.16). The independent risk factors for IVAC were the same as VAC: AKI severity (OR, 2.22; 95% CI, 1.15–4.30), peak %FO within 3 days preceding the VAC (OR, 1.23; 95% CI, 1.03–1.47) and mean PIP (OR, 1.14; 95% CI, 1.02–1.26). A multivariate logistic regression model adjusted for severity of illness using PIM2 score (Table 4) identified peak FOKIS within 3 days as an independent risk factor for VAC (OR, 1.29; CI, 1.13-1.47) and IVAC (OR, 1.26; CI, 1.06-1.49), respectively. The peak FOKIS within 3 days before VAC occurrence could discriminate VAC cases from a matched control, with an area under the receiver operating characteristic curve of 0.74 (95% CI, 0.65–0.83; *p* < 0.001) (Figure 1). When using FOKIS to predict VAC probability, VAC occurrence increased as FOKIS increased; this relationship appears more prominent when the FOKIS is > 7 (Figure 2).

**Table 3:** Multivariate risk factor analysis for ventilator-associated conditions (VAC) and infection-related ventilator-associated complications (IVAC) adjusted for severity of illness score.

**Variable**	**Odds ratio**	**95% CI**	***P*-value**
**VAC EVENT**			
AKI Severity (pRIFLE)	2.15	1.39–3.31	<0.001
PIM 2 score	1.02	0.80–1.31	0.826
Peak daily %FO within 3 days preceding event	1.22	1.08–1.37	0.001
Mean PIP	1.09	1.02–1.16	0.009
**IVAC EVENT**			
AKI Severity (pRIFLE)	2.22	1.15–4.30	0.017
PIM 2 score	1.03	0.76–1.39	0.825
Peak daily %FO within 3 days preceding event	1.23	1.03–1.47	0.017
Mean PIP	1.14	1.02–1.26	0.013

*AKI, Acute Kidney Injury; pRIFLE, Pediatric Risk, Injury, Failure, Loss, End-Stage Renal Disease; PIM 2, Pediatric Index of Mortality; I/O, Intake/output; PIP, Peak inspiratory pressure*.

*The models included pRIFLE score, PIM 2 score, fluid overload variables, mean PIP and mean Paw*.

**Table 4:** Multivariate risk factor analysis of FOKIS and covariates for ventilator-associated conditions (VAC) and infection-related ventilator-associated complications (IVAC) adjusted for severity of illness score.

**Variable**	**Odds ratio**	**95% CI**	***P*-value**
**VAC EVENT**			
Peak FOKIS within 3 days preceding event	1.29	1.13–1.47	<0.001
PIM 2 score	1.11	0.88–1.40	0.451
Mean PIP	1.08	1.02–1.15	0.007
**IVAC EVENT**			
Peak FOKIS within 3 days preceding event	1.26	1.06–1.49	0.006
PIM 2 score	1.07	0.80–1.43	0.629
Mean PIP	1.14	1.04–1.25	0.004

*FOKIS, Fluid Overload and Kidney Injury Score; PIM 2, Pediatric Index of Mortality; PIP, Peak inspiratory pressure*.

*The models included FOKIS variables, PIM 2 score, mean PIP and mean Paw*.

## Discussion

Ventilator-associated events (VAE) are associated with increased morbidity and mortality in both adults and children. Many risk factors are associated with development of VAEs in critically ill children, with AKI and FO among the identified risk factors (8). Our study demonstrates that FOKIS, a kidney injury specific score and its clinical variables are associated with VAE. Our study is the first to identify AKI and severity of FO as possible associated risk factors for VAC using the new pediatric VAC definitions, and IVAC when using CDC's adult definition.

The incidence of VAC in this study was 8.4 per 1,000 ventilator days, higher than 2.9 per 1000 ventilator days reported by Cocoros et al. (15). The higher incidence in our study could be explained by: inclusion of patients on high frequency oscillatory ventilation, likely higher severity of illness score and exclusion of cardiac and neonatal ICU patients in our cohort. We used the CDC's definition for identifying adults with IVAC because validated IVAC criteria for children are not available (19). The incidence of IVAC in this study of 2.2 per 1000 ventilator days was lower than the incidence of 3.6 to 4.7 per 1000 ventilator days reported in critically ill adults (20–22); this could be a reflection of differences in risk factors for VAE in adults compared to children.

The role of AKI and lung dysfunction has been extensively reported in adults, (9–13) and our study our study affirms that AKI is still associated with VAC using the pediatric definitions. As most of the prior studies are retrospective, it has been difficult to argue causation, as a definite chronological sequence between VAE following the onset of AKI has not been established. In our study, as the peak FOKIS score within the 3 days preceding an event is predictive of VAC and IVAC episodes, we propose a possible chronological link between an onset of AKI and VAEs, as FOKIS contains AKI as a sub-score.

Bidirectional organ cross-talk between kidney and lung, with spillover of cytokines and damage associated molecular patterns into circulation mediating inflammation in the distant organ, has been proposed as the link between AKI and propagation of lung injury (23, 24). Increased pulmonary vascular permeability and leukocyte trafficking are also possible causes of lung injury after AKI (10–13). Fluid overload, which frequently accompanies oliguric AKI, can lead to increased venous pressures, which in turn leads to elevated renal venous pressure, compromising renal perfusion (25). Lung injury and increased extracellular lung water associated with AKI could potentiate the risk of infection. In addition, a high incidence of AKI has been reported in adult patients with VAP (12). Prudent attention to the management of mechanical ventilation and lung-kidney interactions is required to address this deleterious organ cross-talk. What is still not clear and requires further study is whether oliguria associated with AKI leads to pulmonary extravascular fluid accumulation and hence increases propensity to VAE, or whether prevention of AKI could modulate inflammatory profile and prevent lung injury.

We found that FO was an independent risk factor for VAC and IVAC. This result is consistent with a previous study by Lewis et al. (6), identifying positive fluid balance as a risk factor for adult's VAC whereas another study in adults demonstrated that depletive fluid management was also helpful in reducing rate of VAP (7). Several reports have demonstrated that FO is a cause of increased morbidity in pediatric patients (4, 26). Specifically, both early FO and net positive fluid balance for each day are found to be associated with increased morbidity (4). In children with acute respiratory distress syndrome, FO causes a decrease in oxygenation and ventilator free days (27). Increased lung water retention is likely an explanation of worsening in oxygenation by causing pulmonary edema which, in turn, may result in VAC. The study by Guess et al. (8) showed that FO was a risk factor for VAC and IVAC. Our study shows these findings remain true when using the pediatric definition. Administration of liberal fluid to patients with respiratory failure could lead to FO with deleterious consequences on oxygenation and length of ventilation. Despite recommendations for conservative fluid strategy, clinicians often administer large amount of fluid in pediatric acute lung injury (28). We demonstrate that FO might be a modifiable risk factor for VAEs, as the peak daily %FO within the 3 days preceding an event is associated with the VAC and IVAC episodes, establishing a timeline. Still remains to be investigated whether fluid restriction or diuretic therapy once an FO threshold is reached will actually prevent VAE episodes.

The FOKIS study by Akcan-Arikan et al. (14) demonstrated that higher FOKIS was associated with morbidity and mortality in PICU. Our study is the first in pediatrics to use a decision-support tool for identifying patients at risk of VAC and/or IVAC. A previous study in adults used a decision-support system to aid diagnosis of VAP (29). This report concluded that timely identification of patients at risk of VAP led to early detection and early prevention of this condition. Our study highlights the utility of FOKIS for detection of patients at risk of VAEs in addition to its usual role of quantitatively scoring renal disease burden in PICU. As FO and nephrotoxic medication exposure could be potentially modifiable risk factors, prospective daily monitoring of FOKIS may lend itself to developing preventive measures and/or quality improvement initiatives to reduce VAEs. We believe the utility of our findings would be optimized in the context of a clinical decision support tool. An interruptive alert that is actionable, by linking to either a care bundle, would be expected to maximize clinical application. This facet of FOKIS remains to be tested and constitutes our overarching goal.

Our study had limitations. First, it was a retrospective study that used data from the FOKIS database. We adjusted our VAC surveillance module which was used in our previous study (3) to identify cases. We reviewed mean airway pressure and FiO_2_ in all patients to identify cases in order to reduce missing data. Second, the FOKIS was designed to assess renal disease burden. Some of its variables may not be associated with development of VAE. Future study for a prospective implementation of the FOKIS should be performed to confirm its benefit and its inclusion in a prevention bundle for VAE.

## Conclusion

This study demonstrated an association of fluid overload status and acute kidney injury in mechanically ventilated children with VAC and IVAC. The FOKIS developed to monitor renal burden was also associated with VAC and IVAC. Further studies should determine the utility of FOKIS as a predictor for VAE.

## Ethics Statement

This study was carried out in accordance with the recommendations of the Institutional review board (IRB) at the Baylor College of Medicine. The protocol was approved by the IRB with a waiver of written informed consent from all subjects.

## Author Contributions

JV conceptualized the study design, collected and analyzed the data, and wrote and edited the manuscript. AA-A and JS contributed to the study design, reviewed and edited the manuscript. JC-B contributed to the study design, analyzed the data, reviewed and edited the manuscript. CK conducted data curation, contributed to data analysis, reviewed and edited the manuscript. ST conceptualized the study design, supervised data collection and analysis, wrote, reviewed, and edited the manuscript.

### Conflict of Interest Statement

The authors declare that the research was conducted in the absence of any commercial or financial relationships that could be construed as a potential conflict of interest.
