# Supplementary material for: Fluid Overload and Kidney Injury Score as a Predictor for Ventilator-Associated Events
Source: Front Pediatr. 2019 May 22;7:204. doi: 10.3389/fped.2019.00204 (PMC6538930; doi:10.3389/fped.2019.00204)
Supplement: Supplementary file 1 [file Table_1.docx]

**Supplementary material**

**Table 1. FOKIS Components**

| **FOKIS CALCULATION** | | |
| --- | --- | --- |
| **pRIFLE Creatinine Criteria** | | **Points** |
| Risk | GFR decrease by 25% | 1 |
| Injury | GFR decrease by 50% | 2 |
| Failure | GFR decrease by 75%  or eCCI<35 ml/min/1.73m^2^ | 3 |
| **pRIFLE Urine Output Criteria** | | **Points** |
| Risk | <0.5 ml/kg/h for 8 hr. | 1 |
| Injury | <0.5 ml/kg/h for 16 hr. | 2 |
| Failure | <0.3 ml/kg/h for 24 hr.  or anuric for 12 hr. | 3 |
| **Fluid Overload Score** | | **Points** |
| Fluid Overload of 15% - <20% | | 1 |
| Fluid Overload of 20% - <25% | | 2 |
| Fluid Overload of 25% - <30% | | 3 |
| Fluid Overload of 30% - <35% | | 4 |
| Fluid Overload of >35% | | 5 |
| **Nephrotoxic Medications Score** | | **Points** |
| Three Nephrotoxic Medications | | 1 |
| Each Additional Nephrotoxic Medication | | +1 |
