# Supplementary material for: Fluid Overload and Kidney Injury Score as a Predictor for Ventilator-Associated Events
Source: Front Pediatr. 2019 May 22;7:204. doi: 10.3389/fped.2019.00204 (PMC6538930; doi:10.3389/fped.2019.00204)
Supplement: Supplementary file 2 [file Table_2.docx]

**Supplementary material**

**Table 2. List of nephrotoxic medications included in FOKIS**

| **Nephrotoxic medication list** |
| --- |
| \| Acetazolamide \| \| --- \| \| Amikacin \| \| Amphotericin \| \| Chlorothiazide \| \| Cisplatin \| \| Cyclosporine \| \| Ethacrynic acid \| \| Furosemide \| \| Gentamicin \| \| Ibuprofen \| \| IV Contrast \| \| Ketorolac \| \| Metolazone \| \| Tacrolimus \| \| Tobramycin \| \| Vancomycin \| |
